# TheraCal LC: From Biochemical and Bioactive Properties to Clinical Applications

**DOI:** 10.1155/2018/3484653

**Published:** 2018-03-26

**Authors:** Naji Ziad Arandi, Tarek Rabi

**Affiliations:** ^1^Department of Conservative Dentistry and Prosthodontics, Faculty of Dentistry, Arab American University, Jenin, State of Palestine; ^2^Department of Conservative Dentistry, Al-Quds University, Jerusalem, State of Palestine

## Abstract

**Background:**

Direct pulp capping is a popular treatment modality among dentists. TheraCal LC is a calcium silicate-based material that is designed as a direct/indirect pulp capping material. The material might be very attractive for clinicians because of its ease of handling. Unlike other calcium silicate-based materials, TheraCal LC is resin-based and does not require any conditioning of the dentine surface. The material can be bonded with different types of adhesives directly after application. There has been considerable research performed on this material since its launching; however, there are no review articles that collates information and data obtained from these studies. This review discusses the various characteristics of the material with the aim of establishing a better understanding for its clinical use.

**Methods:**

A search was conducted using search engines (PubMed and Cochrane databases) in addition to reference mining of the articles that was used to locate other papers. The process of searching for the relevant studies was performed using the keywords pulp protection, pulp capping, TheraCal, and calcium silicates. Only articles in English published in peer-reviewed journals were included in the review.

**Conclusion:**

This review underlines the fact that further *in vitro* and *in vivo* studies are required before TheraCal LC can be used as a direct pulp capping material.

## 1. Introduction

The primary objective of restorative dentistry is to preserve pulpal health of vital teeth. Currently, there is no single pulp protection protocol for clinicians to follow [1, 2]. A direct pulp cap is a procedure in which the exposed vital pulp is treated with a therapeutic material, followed by a base and restoration, to promote healing, to maintain pulp vitality, and to protect the pulp from thermal, chemical, and noxious stimuli [1]. Calcium hydroxide has traditionally been used as the pulp capping material of choice for pulpal exposures in permanent teeth [3]. The effect of calcium hydroxide is the result of the chemical injury caused by the hydroxyl ions released during the hydration reaction into the surrounding environment [2]. Calcium hydroxide stimulates the pulp to defend and repair to form a reparative dentin bridge. It has been reported that 89% of 192 dentin bridges formed by calcium hydroxide cement in monkeys contained tunnel defects that might fail to provide a permanent barrier and a long-term biological seal against bacterial infection [4]. However, the major disadvantage of calcium hydroxide is its high solubility which leads to the disappearance of the material and the formation of defects in reparative dentin underneath the capping material, thereby failing to provide a permanent seal against bacterial invasion [5].

Calcium silicate-based materials are bioactive materials capable of forming apatite by using calcium silicates or calcium aluminates. These materials are also biointeractive [6]; they release ions needed to stimulate dentin bridging. Mineral trioxide aggregate (MTA) is a calcium silicate-based material that is used in direct and indirect pulp capping in primary and permanent teeth. MTA has been reported superior to calcium hydroxide for pulp capping of mechanically exposed human teeth [7–9]. However, MTA exhibits many drawbacks as a capping material such as difficult handling, long setting time, induction of tooth discoloration, and incompatibility with other dental materials when layered [10].

New calcium silicate materials have appeared recently, and among them, Biodentine (Septodont, Saint Maur-des-Fosses, France) is a modified MTA-like material that was introduced to overcome the drawbacks presented by MTA as a pulp capping material. Biodentine is a calcium silicate-based dentin substitute presented as a powder in a capsule composed of tricalcium silicate cement, zirconium oxide, and calcium carbonate. The liquid in the ampule is composed of water, calcium chloride, and a water-based polymer [11]. Biodentine can be applied directly into the prepared cavity as a bioactive bulk dentin substitute that induces dentin bridge formation. Biodentine has been reported to form a mineral-rich interfacial layer and a tag-like structure extending from the interfacial layer to the dentinal tubules that anchors the material micromechanically to the underlying dentine [12]. It has good mechanical properties as well as excellent biocompatibility and bioactive behavior. In addition, it sets in approximately 12 minutes and does not cause tooth discoloration [11]. Brizuela et al. [13] conducted a randomized clinical trial to investigate the outcome of direct pulp capping of permanent young teeth with Biodentine and compare the results with MTA (ProRoot MTA, Dentsply, Maillefer) and calcium hydroxide. The study showed that Biodentine had no failures after 12 months while each of calcium hydroxide and MTA had a 13.6% failure rate after the same time period. However, the study reported that there were no statistically significant differences among the materials studied at different time intervals, that is, 3, 6, and 12 months. Similar results were reported in a split-mouth study by Katge and Patil [14]. The study reported 100% success rate at 6 and 12 months with both MTA and Biodentine when used as direct pulp capping agent in first permanent molars in 7- to 9-year-old children. Nowicka et al. [15] also reported similar clinical results for both MTA and Biodentine in premolars extracted for orthodontic reasons after 6 weeks of follow-up. However, the main drawback with using Biodentine is its water-based chemistry and thus poor bonding as the bond is mainly micromechanical to the overlying resin restoration. To overcome this limitation, a light-curable resin-modified tricalcium silicate (TheraCal LC) was introduced as a pulp capping material.

TheraCal LC is a single paste calcium silicate-based material promoted by the manufacturer for use as a pulp capping agent and as a protective liner for use with restorative materials, cement, or other base materials. This material has been classified as a 4th generation calcium silicate material [16]. According to ISO 9917-2017 part 2 clause 4.1, TheraCal LC is a class 2 cement material “in which the setting reaction of the polymerizable component is light-activated.”

## 2. Search Methodology

An electronic search was conducted in the PubMed and Cochrane databases with appropriate MeSH headings and keywords related to the physical, chemical, sealing ability, biocompatibility, antibacterial properties, clinical applications, drawbacks, and mechanism of action of TheraCal LC. Combinations of the following keywords were used for the identification of the studies to be considered in this review: “TheraCal LC,” “pulp capping,” “vital pulp therapy,” “calcium silicate cements,” “pulp protection,” and “bioactive liners.” To enrich the results, a hand-search was conducted of the last 2 years' worth of issues of the following major endodontic and dental material journals: *International Endodontic Journal; Journal of Endodontics; Dental Materials;* Reference mining of the articles that were identified was used to locate other papers. The process of cross-referencing continued until no new articles were identified. Only articles in English published in peer-reviewed journals were included in the review.

## 3. Review

TheraCal LC is a hybrid material [16]. The original patent sheet stated that TheraCal LC consists of Portland type III cement (45%), fumed silica as a thickening agent (7%), resin (43%), bismuth oxide (3%), and barium sulfate (3%) as radiopaquers [17]. The safety data sheet provided from the manufacturer (dated August 2011) shows that TheraCal LC is mainly composed of Portland cement type III (20–60%), poly(ethylene glycol) dimethacrylate (10–50%), bis-GMA (5–20%), and barium zirconate (1–10%) [18]. However, safety data sheets dated March 2012 show that the material is HEMA-free and composed of Portland cement (30–50%), polyethelyene glycol dimethacrylate (10–30%), and barium zirconate (1–10%) [19]. This was confirmed by the study of Nilsen and Einar [18] which reported that Bis-GMA was not detected in the UPLC-MS analysis of TheraCal LC, despite being listed in the safety data sheet provided by the supplier (dated 2011).

TheraCal LC is claimed to be a hydraulic silicate material that sets by hydration. Hydration is the chemical reaction that leads to the setting of a hydrophilic cement. The setting starts with the contact of the material and water. TheraCal LC does not include water for material hydration. It depends on the water taken up from the environment and its diffusion within the material. Hence, the manufacturer instructions implement placing the material on moist dentin. A study by Camilleri et al. [20] reported that TheraCal LC hydration is incomplete because of the limitation of moisture diffusion from the pulp-dentine complex into the set TheraCal LC.

TheraCal LC has displayed calcium release properties [6, 20–23]. The bioavailability of calcium ions plays a key role in the material-induced proliferation and differentiation of human dental pulp cells and the new formation of mineralized hard tissues. The amount of calcium ions released from TheraCal LC was in the concentration range with potential stimulatory activity for dental pulp and odontoblasts [22, 24]. In a study by Gandolfi et al. [22], TheraCal LC was found to release significantly more calcium than Dycal throughout the tested period (28 days). However, the amount of leached calcium decreased with the time for all the materials (28 days). Another study by Gandolfi et al. stated that the calcium release of ProRoot MTA and TheraCal LC was not marked but constant (no statistically significant changes over time) [21]. This is in contrast to a study by Camilleri et al. which investigated the hydration of TheraCal LC and compared it with Biodentine. The study reported that both materials leached calcium ions in solution. However, TheraCal LC displayed lower calcium ion release and a slower reaction rate than Biodentine [6]. Similarly, the calcium-releasing ability of TheraCal LC was reported to be significantly less than Biodentine [20]. Camilleri et al. [6] attributed the differences in the results due to the different methodologies used in each study. Moreover, Yamamoto et al. [23] and that ProRoot MTA released significantly greater amounts of calcium ions than the TheraCal LC. The study also showed that TheraCal LC does not form calcium hydroxide after setting, although it releases calcium ions and produces calcium apatite on its surface. The absence of calcium hydroxide in set TheraCal LC suggests that calcium ions leached from this material are not in the hydroxide form [6].

On the other hand, the release of hydroxyl ions raises the pH of the surrounding environment and causes irritation of the pulp tissue. This develops superficial necrosis on exposed pulp, provoking mineralization directly against the necrotic zone [25]. An alkaline pH also creates a hostile environment for bacterial survival and proliferation. TheraCal LC has provided a very alkaline (10.66) pH after 3 hours and displayed a nonsignificant reduction in pH (9.85) after 24 hours [25]. Gandolfi et al. [21] reported that TheraCal LC induced lower pH alkalization than the conventional self-setting Dycal did. However, the moderate alkalizing activity of TheraCal LC was constant (9.53 after 3 hours and 8.12 at 28 days) while Dycal maintained the pH 9.81 at 28 days. Yamamoto et al. [23] reported a pH of 9.30 at 5 hours which gradually decreased to 8.4 at 6 hours and 8 at 25 and 144 hours which was significantly lower the pH values recorded for ProRoot MTA at all time periods.

TheraCal LC secures a protective physical lining despite contact with dentinal or pulpal fluids. Its solubility is lower than that of Dycal, ProRoot MTA, Angelus MTA, and Biodentine [21, 22], and its water sorption and porosity is similar to ProRoot MTA and Biodentine, and lower than Angelus MTA [21]. Hence, TheraCal LC may act as a scaffold for reparative dentine formation. Dentinal fluids are absorbed within it, resulting in the release of calcium and hydroxide ions, and the tooth responds to form apatite and a bond, supporting the natural sealing ability of the apatite, plays a crucial role in pulpal protection. TheraCal LC is reported to have an apatite-forming ability [23]. The resultant “apatite coating” plays a key role is dentine repair and mineralization [23]. Its ability to induce the formation of hydroxyapatite-like crystals could contribute to the chemical bond to dentine and provides a biological seal [20, 21]. The sealing ability of MTA, Biodentine, and TheraCal LC was investigated using a confocal laser scanning microscope. The study reported no significant difference in the interfacial microleakage between MTA and Biodentine. However, TheraCal LC exhibited better sealing ability and less interfacial microleakage than the other tested materials [26].

Hydraulic calcium silicate cements are also materials of interest to serve as remineralization agents [27]. TheraCal LC has been developed for indirect (and direct) pulp capping as well. Li et al. [27] compared the remineralization potential of TheraCal LC, Biodentine, and ProRoot MTA. They reported that the hydraulic calcium silicate cements (Biodentine and ProRoot MTA) clearly induced remineralization of artificially demineralized dentine at a higher speed and intensity than the resin-based and resin-free hydraulic calcium silicate cement was attributed to the difference in Ca-ion release kinetics displayed by each material [6, 20, 27]. However, the study reported that the remineralization induced by all the investigated hydraulic calcium silicate cements was incomplete in terms of relative remineralization depth and intensity.

TheraCal LC is a light-cured resin-based material that facilitates the placement of the final restoration as no delay is necessary as shown for water-based pulp capping materials such as Biodentine. The bond strength between restorative and pulp capping materials is important for the success of restorations. TheraCal LC, which is a resin-based material, facilitates layering with composite resin. The effectiveness of layering TheraCal LC with resin composite (Evetric, Ivoclar) bonded with either total-etch adhesives (ExciTe F) or self-etch adhesives (AdheSe One F), and glass ionomer (Fuji IX) was investigated by Meraji and Camilleri [28]. They found that the bond strength between TheraCal LC and composite using the total-etch technique displayed better bond strength values when compared with the self-etch primer. TheraCal LC had higher bond strength values than Biodentine when layered with either composite or glass ionomer cement [28]. However, the study concluded that glass ionomer cements are not indicated for the restoration of teeth in which TheraCal LC is used as a dentine replacement material. Karadas et al. [29] investigated the bond strength of different adhesive agents to TheraCal LC. They reported that etch and rinse adhesives provided the overlying composite restoration with better shear bond strength when compared with self-etch adhesives [29]. The study also showed that two-step self-etch adhesives had higher bond strength values when compared with one-step self-etch adhesives with the exception of the one-step Clearfil S^3^ bond which was attributed to the fact that it contains 10-metacryloxydecyl dihydrogen phosphate (MDP), which has been reported to chemically bond to calcium within the tooth [29, 30]. No correlation was found between the pH of the self-etch adhesives and their bond strength to TheraCal LC [29]. Deepa et al. [31] compared and evaluated the bonding ability of resin composite to three different liners: TheraCal LC, Biodentine, and Fuji II LC using a universal silane-containing adhesive (Single Bond Universal). They found that the bond strength of composite resin to TheraCal LC and Fuji II LC was similar and significantly higher than that of Biodentine following the application of universal adhesive. The bond strength of TheraCal to methacrylate-based composite was significantly higher than that with silorane-based composites and GI cement [32].

TheraCal LC has been referred to as a light-curable MTA-cement [22]. This description has caused some confusion in the literature since there is no light-initiated setting of the Portland cement. The setting reaction depends upon polymerization of the resin component, thus excluding this material from the classification of hygroscopic dental cements that encompasses MTA as well as cements based on bioceramics, calcium silicate, or calcium sulfate [33]. The polymerization of TheraCal LC is associated with low heat generation, and this could reduce adverse pulpal effects when used in pulp capping procedures [34]. TheraCal LC is opaque and “whitish” in color. It should be kept in thin layers so as not to show through composite materials that are very translucent affecting final restoration shade. The manufacturer recommends placing it in 1 mm layers and curing it for 20 seconds. However, Gandolfi et al. [21], reported that the material can be placed in 1.7 mm layers after irradiation for 20 seconds. Nilsen et al. [18] found that TheraCal LC leeched the most camphorquinone of all the investigated materials (Calcimol LC and Ultra-Blend plus), and it was also the only material to leech a co-initiator (DMABEE) which may suggest that the composition of TheraCal LC is not properly suited for light curing.

A pulp capping material should be able to exert antimicrobial activity. Calcium silicate-based materials have better antibacterial activity than calcium hydroxide-based materials [35, 36]. Poggio et al. studied the antimicrobial activity of different pulp capping materials using agar diffusion tests. They found that TheraCal LC had a significantly lower effect on *S. salivarius* and *S. sanguis* when compared with a calcium hydroxide liner (Dycal). However TheraCal LC had similar activity to Dycal when tested against *S. mutans* [35]. The study also demonstrated that the antimicrobial activity of TheraCal LC was similar to the light-cured calcium hydroxide liner Calcimol LC (Voco). Arias-Moliz et al. [36] investigated the antimicrobial activity of leachates from TheraCal LC by means of the minimal inhibitory concentration against *S. mutans*, *S. sobrinus*, and *S. gordonii*. The study reported the absence of antimicrobial activity of the eluents and suggested that the antimicrobial activity might be influenced by the pH.

The cytotoxicity and biocompatibility of a pulp capping material is of particular importance in order to avoid pulp irritation and maintain pulp vitality. Lee et al. [37] conducted a study to evaluate and compare pulpal responses to ProRoot MTA (Dentsply Tulsa Dental, Tulsa, OK), RetroMTA (Meta Biomed Co., Ltd., Seoul, Korea), and TheraCal (BiscoInc, Schamburg, IL) in dog partial pulpotomy cases, whereas a complete dentinal bridge was formed only in 33% of the teeth. They found that TheraCal LC produced the least favorable pulpal responses among the materials used in the study. TheraCal induced an extensive pulp inflammatory reaction in 75% of the lower biocompatibility of the material, which caused a higher degree of inflammation. The study attributed this to the acrylic monomer Bis-GMA present in the material. Poggio et al. [38] studied the cytocompatibility of pulp capping *in vitro* using the Transwell method and reported that TheraCal LC showed very low cytocompatibility. Hebling et al. [39] evaluated the cytotoxic effects of resin-based light-cured liners (TheraCal LC, Vitrabond, Ultra-Blend plus) on pulp cells and reported that all the resin-based liners tested were toxic to the cultured odontoblast-like cells. However, among the tested materials, the light-cured resin-based MTA cement presented the lowest cytopathic effects. Jeanneau et al. [40] studied the consequences of adding resins to tricalcium silicates by investigating TheraCal LC and Biodentine interactions with the dental pulp. Their work showed that TheraCal is toxic to pulp fibroblasts and has a higher inflammatory effect and a lower bioactive potential than Biodentine. Jeanneau and her colleagues stated that upon their preclinical results, TheraCal cannot be recommended for direct pulp capping. Bakhtiar et al. [41] compared the use of TheraCal LC, ProRoot MTA, and Biodentine for partial pulpotomy of sound human third molars. Biodentine performed better than TheraCal LC when used as partial pulpotomy agent and presented the best clinical outcomes. TheraCal LC treatment resulted in pulp disorganization in 66.7% (*n*=9) of the cases beneath the material and in the entire pulp in 22.2% of the cases. Discontinued dentinal bridge was noted in most cases treated with TheraCal. Bakhtiar et al. [41] stated that they do not support the use of TheraCal LC in partial pulpotomy and considered Biodentine and ProRoot MTA more reliable for long-term protection of dental pulp.

In the previous studies [37, 38, 40, 41], the cytotoxicity of TheraCal LC was attributed to its resin components which may remain unpolymerized after contact with pulp tissue. The studies owed the low biocompatibility to the presence of monomers like BisGMA, HEMA, TEGDMA, and UDMA. Nevertheless, it should be noted that the safety data sheet for TheraCal LC does not list BisGMA as a component of TheraCal LC [19]; a fact that was confirmed by the study of Nilsen and Einar [18] which reported that Bis-GMA was not detected in the UPLC-MS analysis of TheraCal LC, despite being listed in the safety data sheet provided by the supplier (dated 2011). This implies that changes in the composition of TheraCal LC might have occurred without the supplier and/or clinicians being notified. Studies on TheraCal LC could, in that case, have been performed with a material of dissimilar composition to the material tested in other studies. Nilsen and Einar [18] investigated the organic composition and eluates of TheraCal LC in relation to its indications and safety data sheets and reported that TheraCal LC contains and elutes several reactive, organic substances that are not declared in the safety data sheets of the material and stated that the use of these materials should be questioned by further *in vitro* and *in vivo* studies.

Nevertheless, TheraCal LC has been reported successful in short term studies. A 2-year *in vivo* study demonstrated that TheraCal LC had a higher success rate (93.3%) for direct pulp capping compared with an antibacterial adhesive system (Protect Bond, Kurary) (83.3%) and glass ionomer cement (Fuji IX, GC) (66.6%) [42]. TheraCal LC had the highest success rates when compared with other test groups treated with self-etch adhesives and Glass Ionomer restorative materials in a 6-month *in vivo* study [43]. Cannon et al. [44] compared the effectiveness of TheraCal LC, pure Portland cement, resin-based calcium hydroxide, or glass ionomer in the healing of bacterially contaminated primate pulps. They found no statistical difference between the groups in regard to pulpal inflammation. However, they reported that the light-cured TheraCal LC groups had significantly more frequent hard tissue bridge formation, a greater thickness of the dentinal bridge and better dentinal bridge qualities than the Glass ionomer and VLC Dycal groups. Gopika et al. [45] compared and evaluated the response of the human pulp following direct pulp capping with TheraCal LC, Septocal LC, and Dycal. Their study found that TheraCal LC and Septocal LC (Calcium hydroxide with hydroxyapatite) cements were as effective as Dycal in inducing the formation of reparative dentin and evoking an inflammatory response. There are a scarcity of studies reporting the outcome of using TheraCal LC in indirect pulp capping procedures. Only one randomized clinical trial reported successful clinical (no pain and absence of sinus tract) and radiographic (no sign of external and internal resorption and presence of bridge) outcomes following the use of MTA and TheraCal LC when used for indirect pulp capping in primary teeth [46].

## 4. Conclusion

Materials with new compositions should be evaluated comprehensively before their clinical application. Future studies should examine whether the lower calcium ion releasing ability, together with the cytotoxic effect due to unpolymerized resin monomers of TheraCal LC has an influence on its biological and clinical performance. Further *in vitro* and *in vivo* studies are required before TheraCal LC can be used as a direct pulp capping material.
